# Feeding Preference and Habitat Association of Defassa Waterbuck (*Kobus ellipsiprymnus defassa)* in Nechisar National Park, Southern Ethiopia

**DOI:** 10.1155/2021/7498662

**Published:** 2021-10-18

**Authors:** Gatriay Tut Deng, Birtukan Tsegaye Demisse

**Affiliations:** ^1^Mekdela Amba University, Zoological Science, Tulawlia, P.O. Box 32, Ethiopia; ^2^Injibara University, Zoological Science, Injibara, P.O. Box 40, Ethiopia

## Abstract

This study investigated feeding preference and habitat association of waterbucks in Nechisar National Park from November 2016 to August 2017 by total count and direct observation methods. From this survey, 20 plant species were observed to be the food items consumed by waterbucks. Waterbucks were mostly grazers than browser. The plant species consumed by waterbucks was highly significant between seasons (*χ*^2^ = 121.34, df = 1, *p* < 0.05). Out of 20 total plant species consumed, annual grass (11.5%), *Leersia virginica* (8.4%), and *Cynodon dactylon* (8.4%) were the most frequently consumed food items, but *Tamarindus indica* (2.9%), *Balanites aegyptica* (3.3%), and *Acacia polycantha* (2.36%) were the least consumed food items. There was significant difference between plant species consumed during both seasons (*χ*^2^ = 177.67, df = 19, *p* < 0.05). The Shannon–Wiener diversity index result revealed that there were different varieties of food items for waterbucks in dry season (H' = 2.9) than in wet season (H' = 2.6). Young leaves comprised the largest proportion of plant parts consumed by waterbucks compared to others. There was a highly significant difference in feeding plant parts in both seasons (*χ*^2^ = 88.5, df = 7, *p* < 0.05). Waterbucks spent more time on feeding in the morning and late afternoon during both seasons. The total number of waterbucks in each habitat varied due to food availability in different seasons. Different conservation measures should be taken against waterbuck reduction and create appropriate environment for waterbuck.

## 1. Introduction

Waterbuck is one of the large-sized mammals, weighing about 250–270 kg and are bright reddish fur color antelopes [1]. It is an even-toed ungulate (Artiodactyla) belonging to family Bovidae in the genus *Kobus* of African Artiodactyle [2]. It is different from other antelopes with special long legs and fur [3]. Based on genetic evidence, there are two distinct subspecies of waterbucks recognized in Africa, namely, the common waterbuck (*Kobus ellipsiprymnus ellipsiprymnus*) and the Defassa waterbuck (*Kobus ellipsiprymnus defassa)* [4, 5]. It has been eliminated widely, but survives in many protected areas populated by human [2]. They are solitary and have social diurnal behavior. It has been revealed that adult males defend core areas of their habitat as their home grounds [6].

Waterbucks are known to be grazers, principally feeding on annual grass species. However, they undergo occasional shift in their diet when grasses are insufficient and become browsers. Their diet includes leaves, twigs, shoot, seeds, fruits, stem, and grass as well as flowers [7]. According to IUCN 3.1 [8], the presence of different vegetation communities in a given habitat support different resources, resulting in high population density of the animals inhabiting the area.

Waterbucks are found mainly in water-rich habitat within the savanna ecosystem; they are adapted to moist savanna ecosystems with a permanent water supply and also prefer open habitats with a short to medium height vegetations for grazing [9]. They inhabit the savannah region of sub-Saharan Africa extending from South Africa north to Ethiopia and South Sudan, west to Senegal. In Ethiopia, they live in west of the Great Rift Valley where there are many major rivers, lakes, and wetlands. They are hardly seen in arid areas and in high altitudinal areas [9]. They occupy the plains during the dry season and reside in woodlands during the wet season [10]. They are territorial and migratory moving up to 1.8 km distance far from water sources for search of food.

The food quality and availability is undoubtedly one of the limitations to the population dynamics of ungulates [11, 12]. In African savannah ecosystems, the mortality brought either by food shortage or by a significant decrease in food quality can even exceed the deaths caused by the various predators [13].

The studies on waterbucks in Ethiopia are scant. Moreover, the feeding and habitat preference of waterbuck is not previously studied in the Nechisar National Park. Therefore, the present study was undertaken to investigate the feeding preference and habitat association of defassa waterbucks in the study area.

## 2. Materials and Methods

### 2.1. Description of the Study Area

**Figure 1:**
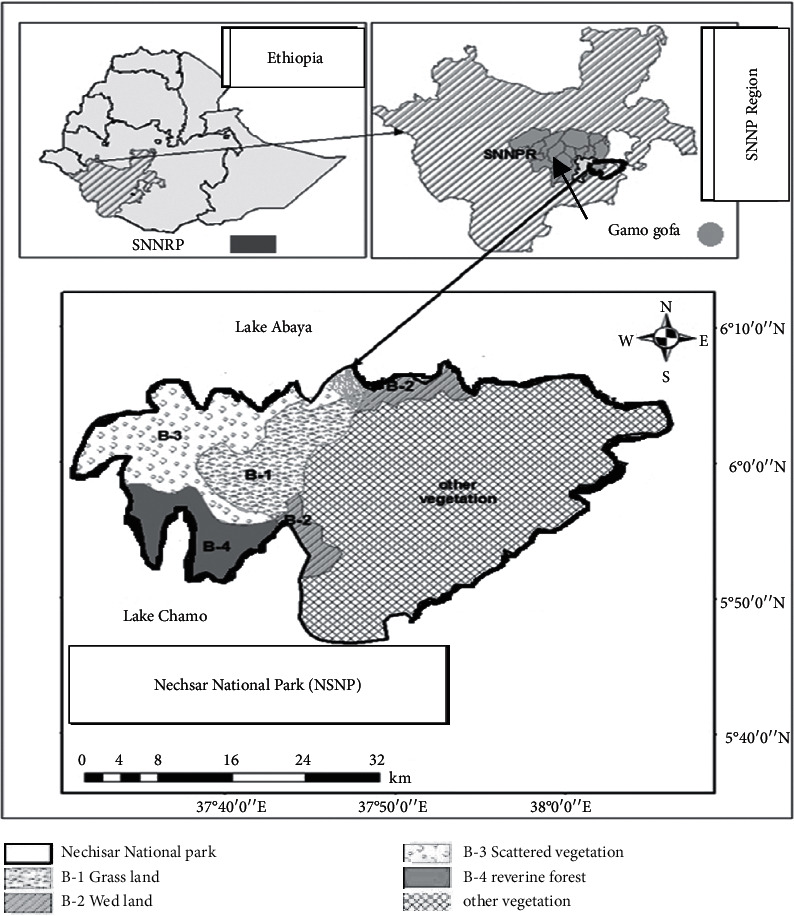
Location of the study area.

The Nechisar National Park (NSNP) is located 510 km south of Addis Ababa near the town of Arba Minch (Figure 1). The NSNP is found at the center of Ethiopian Rift Valley floor between lakes Abaya and Chamo and covers about an area of 514 km^2^ [14]. From the total area of the park, water bodies cover 78 km^2^ with a diversity of forest, grassland, open woodland, and fresh water habitat [14, 15]. The NSNP is located at 5° 51ʹ to 6° 10ʹ N latitude and 37° 32ʹ to 38° 48ʹ E longitude within the elevation range from 1,100 up to 1,600 meter above sea level [16]. A maximum and minimum annual temperature is 30.52°C and 17.3°C, respectively, and the mean annual rainfall is 919.08 mm [17].

### 2.2. Materials and Methods

Before starting the actual data collection, a reconnaissance survey was conducted to gather information on location, fauna, flora, topography, climate, infrastructure, and approximate size of the core habitat of waterbucks. Furthermore, for the study purpose, the study area (514 km^2^) was subdivided into different habitat types (bocks) as grassland with an estimated area of 57 km^2^, wetland covering an estimated area of 21 km^2^, bush land with an area of 50 km^2^, and riverine forest having an estimated area of 78 km^2^. The actual data collection was carried out from January to March and from June to August 2017 to cover both dry and wet seasons, respectively. Each habitat was surveyed two times in each four field visit in both seasons. Throughout the study, data collection took place early in the mornings from 6 : 00–9 : 00 and late afternoon from 16 : 00–18 : 00 in both seasons where the animals are active [18].

During the actual survey, the data collectors were assigned in each habitat to observe the utilized vegetation type, and number of individual waterbucks in each habitat types was recorded. The observations of feeding preference were carried out on foot from a hidden position, and animals were watched with binoculars from a distance varying between 20 m and 50 m for 10 minutes to avoid missing during counting period. If the animals were disturbed, behavioral recording was delayed until they appeared to ignore the observer. Plant samples which the animals were feeding on were taken immediately after the observed animals moved away from the feeding spot. Then, the types of plant species and/or plant parts which the study animals were observed to feed on were collected and identified to measure the feeding preference of waterbucks during both the dry and the wet seasons [19]. At the same time, the habitat where the animals were observed and the season were also recorded in order to estimate the habitat association of the study animals.

The diversity of food items consumed by waterbucks in each season was also investigated. High value of this index indicates that there are different varieties of food items available for waterbucks. Shannon diversity was computed by using the following formula:

(1) $$\begin{array}{c} H^{\prime }=-\sum_{i=1}^{S}\text{pi}*\text{lnpi}, \end{array}$$

where

 
**P*i*** = the proportion of species “*i*”
 
**ln** = natural logarithm
 
**S** = species richness

### 2.3. Data Analysis

The data collected from the study were analyzed using SPSS version 20. Descriptive statistics was used to compare the population size of waterbucks in each habitat type during both seasons. Chi-square test was used to test the statistical difference of seasonal population status; the mean difference in population size and habitat association (habitat type, vegetation type, and habitat utilization) of waterbuck during the wet and dry season; the habitat association of waterbucks; the variation of population size within each habitat; and the seasonal differences in food items in each habitat. In addition, comparison of food items across seasons in each habitat type was done using one-way ANOVA.

## 3. Results

### 3.1. Habitat Association

The population of Defassa waterbucks in the Nechisar National Park was estimated to be 112, with 104.51 and 118.22 individuals in dry and wet seasons, respectively (Table 1). The study result revealed that the highest number of waterbucks was recorded in riverine forest habitat in dry season, whereas the least number was recorded in the bush land habitat in the same season. While during the wet season, the highest number of study animal was recorded in grassland and the least number in wetland. The variation in the number of waterbuck observed in different habitats types was statistically significant within seasons (*χ*^2^ = 15.97, df = 3, *p* < 0.05).

The habitat preference of the study animal was highly associated with the presence of plant food item. Therefore, grassland is the most preferred habitat type followed by bush land habitat. In contrast, the least numbers of waterbucks were recorded in the riverine forest relative to other habitat types. The type of plant food items consumed by waterbucks was statistically significant between each habitat type (*f*_3,930_ = 31.5 *p* < 0.05). The seasonal distribution of waterbucks in the study area is summarized in Table 2.

### 3.2. Feeding Preference of Waterbuck

**Figure 2:**
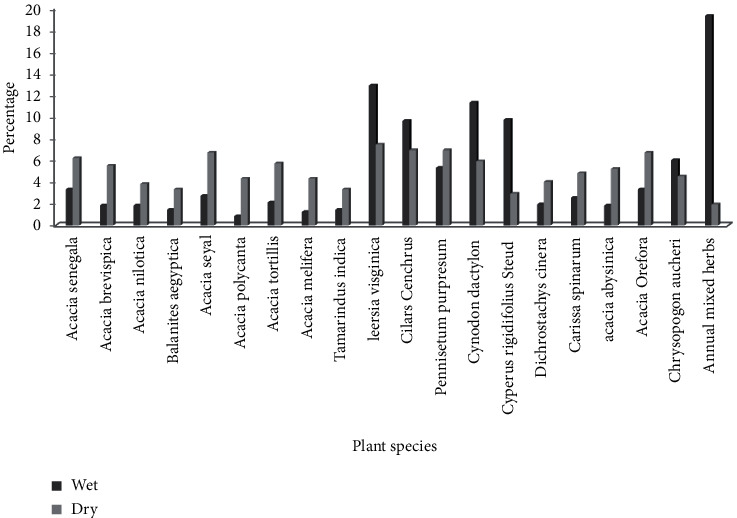
Number of plant species consumed by waterbucks during the wet and dry seasons.

In the present study, 20 plant species which were observed to be consumed by Defassa waterbucks were recorded and identified into 6 families. During the whole study period, many food items were recorded in the wet season (54.9%) than those recorded in the dry season (45.1%). Among the 20 plant species documented to be consumed by waterbucks, the top three plants, namely, annual grass (11.5%), *Cynodon dactylon* (8.4%), and *Leersia virginica* (8.4%), were the most frequently consumed food items. On the contrary, *Tamarindus indica* (2.9%), *Balanites aegyptica* (3.3%), and *Acacia polycantha* (2.36%) were the least consumed food items. There was a highly significant difference in plant species consumed between dry and wet seasons (*χ*^2^ = 177.67, df = 19, *p* < 0.05). The summary of plants species recorded as food items for waterbucks during the study period is presented in Figure 2.

**Figure 3:**
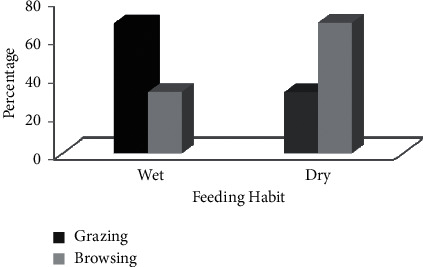
Grazing and browsing habit of waterbucks during wet and dry seasons.

Waterbuck browsed 30.75% of their food during the dry season and 17.55% during the wet seasons and grazed 37.36% during the wet season and 14.34% during the dry season. Waterbuck is grazing more frequently than browsing in the wet season, and grazing is decreased over the dry season (Figure 3). Therefore, there was a high significant difference in the proportions of time spent by waterbucks in grazing and browsing between seasons (*χ*^2^ = 121.34 df = 1, *p* < 0.05).

Waterbucks feed on different plant species within different age groups, namely, adult, subadult, and unidentified sex in different frequencies in each season. Adult waterbucks were active in searching for food and mostly feed on almost all plant species. Subadult and unidentified sex feed most of the time on annual herbs and grass species in both seasons. However, subadult as compared to unidentified sex sometimes feed on other plant species in addition to annual herbs. There was no significant difference in the feeding frequency between plant species consumed among different age groups during both seasons (*f*_2,931_ = 0.21, *p* < 0.05).

**Figure 4:**
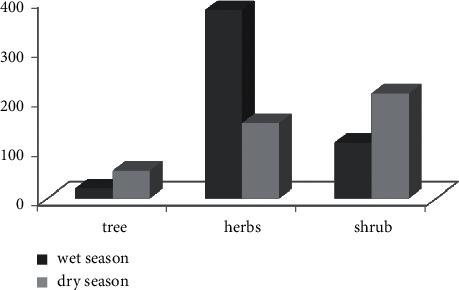
Percentage of plant life form consumed by waterbuck during wet and dry seasons.

Trees, herbs, and shrubs were the principal food items consumed by waterbucks in both seasons. On average, 74.1% and 36.3% herbs were the most frequently consumed life form, and 5.7% and 15.2% trees were the least frequently consumed life form in wet and dry seasons, respectively (Figure 4). Therefore, there was a high significant difference in the frequency of feeding on different life forms during both seasons (*χ*^2^ = 136.14, df = 2, *p* < 0.05).

**Figure 5:**
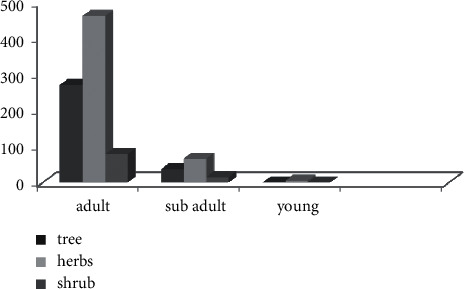
Plant life form consumed by waterbucks within age group.

Based on age structure, subadults and young waterbucks frequently prefer herbs, but adults commonly feed on trees and shrubs in addition to herbs. Therefore, according to the data, there was no significant difference in feeding on different plant life forms among age groups during both seasons (*f*_2,931_ = 0.69, *p* > 0.05) (Figure 5).

## 4. Plant Food Items Diversity

The Shannon–Wiener diversity index result revealed that there were different varieties of food items for waterbucks in dry season (*H*′ = 2.9) than in wet season (*H*′ = 2.6).

### 4.1. Plant Part

**Figure 6:**
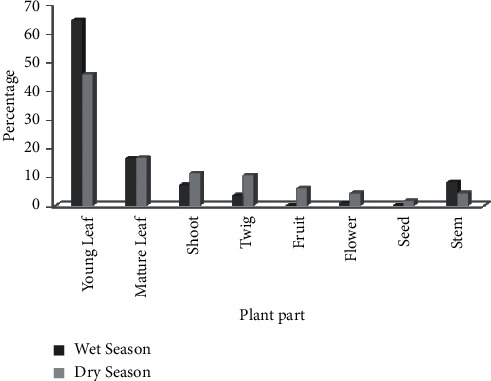
Percentage of plant part consumed by waterbuck during wet and dry seasons.

During the whole study period, a total of 8 plant parts were identified to be consumed by the waterbucks. The study animal (waterbucks) was observed to feed frequently on the three plant parts, namely, the young leaves (55.9%), mature leaves (17.6%), and shoots (8.9%) in both seasons (Figure 6). Throughout the whole study, waterbucks feed most frequently on young leaves as compared to other plants' parts, but in wet seasons, they hardly consume fruit and seed. Therefore, there was a high significant difference in the frequency of feeding on different plant parts during both seasons (*χ*^2^ = 88.5, df = 7, *p* < 0.05).

Based on age structure, subadults and young ones most frequently consumed young leaves, but adults feed on other plant parts in addition to young leaves. There was a high significant difference in the feeding frequency of plant part consumed among three age groups (*χ*^2^ = 64.6, df = 14, *p* < 0.05) (Table 3).

## 5. Discussion

The composition of plant species utilized by the waterbucks as food items in NSNP is high during the wet season and drops in dry season. When the number of plant species increase, it permits increase in the diversity of feeding niches, and it might be the reason for the increasing number of waterbucks in the wet season. Waterbucks tend to become less active and spend more time in bush land during the dry season, which reduces the probability of visibility during the census time [15].

Based on the current result, the waterbucks preferred grassland habitat in wet season than dry season. The low number of waterbucks during the dry season in grassland and bush land habitats may be attributed to the intense human disturbances on the study animal (waterbucks), whereby the waterbucks hide from observers, similar to foraging and behavior of Oribi (*Ourebia ourebia*) in the Senkele Swayne's Hartebeest Sanctuary [20]. Food quality and quantity of waterbuck change seasonally as habitat change during the wet and dry season. It is also an indication of the quality of the habitat favored for them due to their selective feeding behavior to get more preferable forage similar to the finding of Prins H. *et al.* [21], on niche segregation of three small bovid species in southern Mozambique. The biomass of the grass species reaches the top level in grassland during the wet season and declines during the dry season. This decline implies low food availability as the grass species composing large proportion of waterbuck's diet become less abundant and leads to poor diet quality in dry season. Habitat utilization often determines the availability of vegetative covers, food and rich plant species availability resulting in high animal density, and biomass in the area [22, 23].

In the present study, higher number of waterbucks was recorded in riverine forest, bush land, and wetland during the dry season than that of the wet season. This might be because the grasses were more abundant around the riverine forest and wetland habitat during the dry season. Moreover, feeding in the bush land vegetation may help them to overcome the heat and to conserve water loss in the hottest season of the year.

Out of overall plant species, the most preferred diet for waterbuck was annual grasses (11.5%). This adaptive feeding style most probably contributed to the pronounced seasonality in foraging behavior. This study showed that the number of plant species found in the diet of waterbuck in each season varied slightly and feed on different plants parts, namely, tree, shrubs, and herbs [24]. During the rainy season, it was difficult to identify shrubs and herbs consumed by waterbucks due to their various characters so that they were grouped as mixed grasses. This finding is related to the feeding ecology of Grant's gazelle in Abijata-Shalla Lakes National Park reported by Mesele and Afework [25].

Waterbucks feed on leaves, small shoots and fruits like other grazers, a finding similar to the study on trend of water dependent grazers Buffalo and Waterbuck in the Kenya-Tanzania Border land reported by Okello M. *et al.* [1]. Grasses were the most preferred forage types of waterbucks. However, trees were browsed more frequently than dry grasses in dry season. Since grasses species are annual, they dry up in dry season and the waterbucks face problems associated with food shortage and quality decline. In order to cope with such problems, they manage to change their feeding habit from grazer to browser based on the available food types [6, 26]. During wet seasons, green parts of the plants are more nutritious with high moisture content and easily be digested due to low fiber content as opposed to the case in dry season [5], Young leaves constituted the largest proportion of the animal's diet as they are easily digestible and assimilated while, seed and fruit were least consumed plant parts [27]. In the present study, fruits and seeds were more abundant in the dry season compared to the wet season, a similar finding reported by Dereje. et al. [19]. Based on age structure, adult waterbucks were more active in searching for food than subadults and young ones. Subadult and young ones mainly preferred to feed on herbs, but adults feed on trees and shrubs in addition to herbs. The result of the current study shows that waterbucks are mixed feeders grazing on grass species and browsing on shrubs and herbs to survive environmental change.

## 6. Conclusions and Recommendations

### 6.1. Conclusion

Waterbucks were observed to consume varieties of a plant part throughout the study period. Mostly they spend much time in feeding on young leaves. The most preferred waterbuck's diet is perennial, annual plants, grass species, and leaves during the dry season. Waterbucks alternate between grazers and browsers. However, they mostly feed as grazers. Most of the times, subadults and young ones mainly preferred young leaves as their principal diet. To survive environmental change, waterbucks also change their feeding style from leaves part to other plant parts. Waterbucks consumed different plants in different habitat types during wet and dry seasons. Grassland has more abundant food items for waterbucks in the study area. Higher number of waterbucks was recorded in grassland, riverine forest, bush land, and wetland. Therefore, these habitats are said to be most preferred habitats for waterbucks.

### 6.2. Recommendations

The conservation of Defassa waterbuck (IUCN status: Near Threatened) in the future cannot be considered secure in the long term with the present wildlife management. The population status of waterbuck species has declined in the Nechisar National Park, mainly due to adverse human activities. The further destruction of habitats, livestock grazing, and bush encroachment were the observed serious problems in the area.

Based on the present study, the following recommendations are suggested.

- To reduce such threats, immediate conservation measures should be taken for the survival of waterbuck and other wildlife in the National Park.
- Effective conservation measures should be carried out through an extension work to create public awareness among the local community.
- Local community should be aware about the ecological, economic, and social values.
- The sharing of benefits with the communities living adjacent to the park will reduce conflicts between wildlife managers and local communities.
- Local communities should be immediately involved in designing, planning, implementation, and evaluation of the wildlife conservation program. Implementation of rural development should be designed to move the local people to the buffer zone to reduce human activities in the park.
- Buffer zones should be established to reduce the movement of wildlife from crop raiding outside the national park.
- Detailed study investigating the fecal matter is needed to further report the food items that might not have been found through observation.

## Figures and Tables

**Table 1:** Population estimate of waterbuck in the Nechisar National Park (mean ± SD).

Seasonal variation in waterbuck's population	Seasons		
	Wet season	Dry season	Mean
	118.22 ± 9.60	104.51 ± 5.79	112 ± 3.98

**Table 2:** Seasonal distribution of individual waterbucks in each habitat.

Age and sex	Seasons									
	Wet season					Dry season				
	Grassland (B1)	Wetland (B2)	Bush land (B3)	Riverine forest (B4)	Total	Grassland (B1)	Wetland (B2)	Bush land (B3)	Riverine forest (B4)	Total
Adult male	9	4	6	5	24	4	6	2	4	16
Adult female	12	2	10	3	27	4	8	4	15	31
Subadult male	3	2	1	1	7	2	1	3	2	9
Subadult female	4	0	3	2	9	4	0	0	1	5
Young	1	0	1	0	2	0	0	0	1	1
**Total**	**29**	**8**	**21**	**11**	**69**	**14**	**15**	**9**	**23**	**61**

**Total individual waterbucks observed in the whole study period**									**130**	

**Table 3:** Observation of plant part consumed by each age groups.

Age structure	Plant parts								Total
	Young leaves	Mature leaves	Shoot	Twig	Fruit	Flower	Seed	Stem	
Adult	49.5	17.02	6.64	5.89	1.93	1.82	0.75	3.43	86.95
Subadult	6.1	0.53	2.25	0.75	0.75	0.53	0	1.61	12.52
Young	0.32	0	0	0	0	0	0	0.21	0.535
Total	55.88	17.55	8.89	6.64	2.68	2.35	0.75	5.25	100

## Data Availability

The data used to support the findings of this study are available from the corresponding author upon request.
